# Practice Patterns of Hip Flexion Assist Orthosis Use in Multiple Sclerosis: A Retrospective Community-Based Analysis

**DOI:** 10.1016/j.arrct.2026.100640

**Published:** 2026-05-25

**Authors:** Christopher R. Bhatla, Rajiv N. Reebye

**Affiliations:** a MD Undergraduate Program, Faculty of Medicine, University of British Columbia, Vancouver, BC, Canada; b Division of Physical Medicine and Rehabilitation, Temerty Faculty of Medicine, University of Toronto, Toronto, ON, Canada; c Division of Physical Medicine and Rehabilitation, Faculty of Medicine, University of British Columbia, Vancouver, BC, Canada

**Keywords:** Gait, Hip joint, Multiple sclerosis, Orthotic devices, Rehabilitation, Walking

## Abstract

**Objective:**

To identify patterns of hip flexion assist orthosis (HFAO) use among ambulatory patients with multiple sclerosis (MS) and to assess factors influencing device adoption and discontinuation.

**Design:**

Retrospective chart review.

**Setting:**

Outpatient community physiatry clinic in New Westminster, British Columbia.

**Participants:**

Fifty-four ambulatory patients with MS who were previously assessed for HFAO use between November 2017 and October 2023. Patients were 35-92 years old, able to participate in gait evaluation, and had documented physical examinations. Patients with nonambulatory status or missing motor strength data were excluded. Participants were categorized into groups based on HFAO outcomes: regular use, no follow-up postpurchase, discontinued use, declined HFAO, or unsuccessful HFAO assessment.

**Interventions:**

Not applicable.

**Main Outcome Measures:**

Rates of HFAO use and discontinuation, hip flexor and knee extensor Medical Research Council (MRC) strength scores, lower-limb spasticity using the Modified Ashworth Scale (MAS), and qualitative factors associated with device adoption and nonuse.

**Results:**

Of 54 patients assessed, 48 were recommended an HFAO. Forty obtained a device, and 19 used it regularly. Thirteen patients discontinued use after a mean of 20 months, primarily due to pain/discomfort (38%) and device bulkiness (31%). Discontinuation was associated with higher lower-limb spasticity (mean MAS 1.5 vs 0.77, *P*=.011). Cost was the primary barrier to adoption (75%). Most patients exhibited hip flexor weakness (MRC=1-3) and relatively preserved knee extensor strength (MRC≥3).

**Conclusions:**

This study identifies clinical factors associated with HFAO use and discontinuation in ambulatory patients with MS, including hip flexion MRC scores of 1-3, knee extension MRC scores ≥3, and spasticity MAS scores <2. Comprehensive pre-prescription assessment, including pain screening and gait evaluation with and without a device, is recommended to optimize patient selection.

## Highlights

• Hip flexion assist orthoses (HFAO) can aid ambulation in people with multiple sclerosis.
• HFAO use is associated with weak hip flexors and relatively preserved knee extensors.
• Low spasticity is also associated with long-term continued use.
• Barriers to adoption and continued use include cost, discomfort, and bulkiness.
• Comprehensive pre-prescription evaluation is recommended to optimize outcomes.

Multiple sclerosis (MS) is an inflammatory disease characterized by demyelination and axonal degeneration in the brain and spinal cord. It presents a wide range of clinical features depending on the location of lesions. Common effects of MS include extremity weakness, spasticity, and impaired gait caused by pyramidal tract lesions.^1^, ^2^, ^3^ In the case of focal weakness, orthoses can be used to compensate.^3^^,^^4^ Ankle-foot orthoses are commonly used for dorsiflexion weakness (foot drop). In the case of hip flexor weakness, a hip flexion assist orthosis (HFAO) can be used.^2^^,^^3^^,^^5^

An HFAO is designed to help initiate hip flexion during the swing phase and improve a patient’s ability to ambulate.^5^ These devices consist of a hip belt with an elastic band system that provides an assistive force at the hip during the swing phase. The elastic bands commonly cross the knee and anchor to the foot (providing a secondary dorsiflexion effect at the ankle), although alternative designs may attach to the thigh instead. Commercially available models weigh between 0.5 and 0.75 kg and cost approximately CAD $400-$900.

Currently, there are no widely accepted guidelines for HFAO use. A small number of studies have evaluated HFAOs without directly addressing prescription guidelines. Sutliff et al^6^ conducted a prospective trial of 21 ambulatory patients with MS and unilateral hip flexor weakness, demonstrating significant improvements in lower-extremity strength and gait performance measures at 8 and 12 weeks. Neuman et al^7^ further confirmed biomechanical benefits in a small exploratory study, showing that HFAO use increased peak hip flexion angle and improved hip energetics toward more normative gait patterns. More recently, Prada et al^8^ evaluated 50 patients with MS during their first use of a passive hip flexion exoskeleton, finding no significant improvement in objective gait measures but a positive or neutral global perceived effect in 72% of participants, underscoring the importance of incorporating patient-reported outcomes into HFAO prescription decisions. Additional studies have investigated HFAO use in patients with hemiparesis after stroke.^9^, ^10^, ^11^ However, no studies have assessed which patients with MS should be prescribed HFAOs.

Typically, an HFAO prescription is guided by practitioners’ experience and patients’ preferences. Symptom variability is a hallmark of MS, particularly in relapsing-remitting disease, and can affect a patient's day-to-day functional capacity.^1^ While this variability complicates decision-making, the absence of standardized criteria means that key clinical features may not be systematically considered during prescription. By determining current patterns of HFAO use, this study aimed to better characterize which patients are most likely to benefit from the device, with the goal of informing future clinical practice.

Furthermore, assistive technology abandonment is common in patients with MS, with up to 37% of devices being abandoned within 3 years.^12^ Studies specific to HFAOs have followed patients for only a few weeks, without evaluating their long-term use. This study hypothesized that HFAO use would mirror the high early abandomnent rates observed in other assistive devices and sought to better understand why certain patients who could benefit from an HFAO are not using one. This research may then provide a starting point for future work to increase rates of long-term HFAO use and improve overall patient function.

## Methods

A retrospective chart review was conducted at a rehabilitation medicine clinic in New Westminster, British Columbia. In this interdisciplinary clinic, patients are first seen by a physiatrist with more than 18 years of clinical experience managing MS. If the physiatrist believes the patient may benefit from an HFAO, the patient undergoes an HFAO assessment during the same appointment, with both the physiatrist and an orthotist present. This assessment involves donning a sample HFAO and completing a series of straight walks and turns. If the HFAO is deemed beneficial and the patient wishes to proceed, it is prescribed. Patients then typically purchase an HFAO at the orthotics practice of the same orthotist (although they may choose another practice if they wish), with no remuneration provided to the physiatrist or the rehabilitation medicine clinic. HFAOs are not covered by British Columbia’s provincial health care plan. Thus, patients either pay out of pocket or have coverage through additional insurance.

### Study design and participants

The chart review examined patients who were assessed by the clinic physiatrist for consideration of an HFAO between November 1, 2017, and October 31, 2023.

Inclusion criteria were as follows: (1) patients diagnosed with MS; (2) patients aged ≥15 years; (3) cognitively able to answer questions and consent to HFAO use; (4) able to ambulate indoors independently or with 1-person assistance; (5) patients with at least 1 documented in-person assessment during the date range of the charts accessed (November 1, 2017, to October 31, 2023); (6) patient encounters were in English or with an interpreter.

Nonambulatory patients and those lacking essential chart data, such as motor strength scores on physical examination, were excluded. Fifty-four patients met all inclusion/exclusion criteria and were selected for chart review. Each patient was assigned a unique identifier unrelated to any personal identifiers.

### Data extraction

The following data were extracted from each chart (for all dates, only the month and year were collected): (1) sex; (2) year of birth; (3) MS diagnosis; (4) date of MS diagnosis; (5) date of HFAO in-person assessment; (6) date of HFAO prescription (if applicable); (7) date HFAO discontinued (if applicable); (8) Medical Research Council (MRC) muscle strength at the time of HFAO assessment. Hip flexion, knee extension, and ankle dorsiflexion were the only muscle groups consistently reported. (9) Lower-limb spasticity assessment using the Modified Ashworth Scale (MAS). Spasticity documentation varied across charts; some recorded a single limb-level MAS score (eg, “left lower limb MAS 1”), while others specified individual muscle groups. In both cases, the highest recorded MAS score for any lower-limb muscle group was used as the summary measure for each patient; (10) use of other gait aids and orthoses; (11) functional status of self-reported activities of daily living (ADLs) and instrumental ADLs.

During chart extraction, the following qualitative data were captured, with all personal identifiers removed: (1) for each patient who discontinued HFAO use, text from the original clinical notes detailing the reason for discontinuation was extracted from the medical charts; (2) any additional text in the notes from the physiatrist and orthotist related to HFAO use was extracted. This included descriptions of patients’ gait with and without an HFAO.

### Analysis

For analysis, patients were divided into 5 groups based on their assessment results and subsequent HFAO use (the text in parentheses represents the shorthand terminology used for the remainder of this paper): (1) currently using an HFAO regularly (Using HFAO); (2) purchased an HFAO but not yet seen in follow-up (purchased, no follow-up); (3) purchased an HFAO but discontinued use (discontinued); (4) chose not to purchase an HFAO, despite a recommendation by the physiatrist after assessment (declined HFAO); (5) HFAO was not recommended by the physiatrist after assessment (unsuccessful assessment).

For patients who did not purchase an HFAO or discontinued its use, a descriptive content analysis was used to categorize the reasons for nonuse or discontinuation. First, the extracted text for each patient who discontinued HFAO use was reviewed. Then, categories were developed to encompass all identified reasons, and each patient was assigned to a corresponding category.

### Statistical analysis

To assess whether lower-limb strength or spasticity differed across outcome groups, MRC and MAS scores were compared using the Kruskal-Wallis test at the 95% confidence level. A value of 1.5 was assigned to MAS scores of 1+. For significant Kruskal-Wallis differences, the Dunn test with Bonferroni correction was used for post hoc pairwise comparisons.

### Ethics and reporting

This study received ethics approval from the University of British Columbia Clinical Research Ethics Board (H23-02793). Informed consent was waived due to the study’s retrospective design and the use of deidentified clinical data. The study was conducted and reported in accordance with the Strengthening the Reporting of Observational Studies in Epidemiology guidelines for observational studies.

## Results

Table 1 shows the demographic information and functional status of the study participants. Patients were aged 35 to 92 years (mean=59); the women-to-men ratio was approximately 3:2. Eighty-five percent of patients were independent in all ADLs and instrumental ADLs, a proportion that was uniform across outcome groups. Eighty percent of patients used 1 or more gait aids, including canes, poles, crutches, and walkers. Thirty-seven percent had an active, concurrent lower-limb orthosis at the time of assessment, most commonly a unilateral ankle-foot orthosis or a Dictus brace, with 2 patients using bilateral devices. Expanded Disability Status Scale scores and ethnic background were not included because this information was not consistently available in the review charts.

**Table 1:** Demographics of study participants

Characeristic		Using HFAO	Purchased, No Follow-Up	Discontinued	Declined HFAO	Unsuccessful Assessment	Overall
No. of participants		19	8	13	8	6	54
Age (y), mean		58	54	61	55	64	59
Sex, M/W		6/13	5/3	2/11	5/3	3/3	21/33
Diagnosis	Primary progressive	4	2	2	4	3	15
	Secondary progressive	5	3	2	3	1	14
	Relapsing remitting	4	2	3	0	0	9
	Not specified	6	1	6	1	2	16
Time since diagnosis (y), mean		22	20	24	16	17	21
Gait aids/orthoses at the time of HFAO assessment	Using a walker	8	1	9	4	1	23
	Using a cane/poles/crutches	12	4	7	4	4	31
	Using any gait aid (includes the above)	15	5	11	6	6	43
	Using orthosis	7	2	7	2	2	20
ADLs/IADLs	Independent for ADLs and IADLs	16	8	11	7	4	46
	Independent for ADLs, assistance for IADLs	2	0	2	1	2	7
	Assistance for ADLs and IADLs	1	0	0	0	0	1

Abbreviations: IADL, instrumental activity of daily living.

### Use patterns and descriptive content analysis

Of the 54 patients who underwent an HFAO assessment, 48 were recommended an HFAO by the physiatrist, comprising 43 unilateral and 5 bilateral HFAOs. An HFAO was not recommended for 6 patients. In 4 of these, the HFAO worsened their gait. Although the exact changes in their gait were not clearly documented, qualitative comments suggested that forces acting on the HFAO hip belt caused balance difficulties for these patients. For 2 patients, the HFAO was not found to be helpful.

Of the 48 patients for whom an HFAO was recommended, 40 followed up with the orthotist to procure a device, while 8 did not. Of these 8 patients, 6 cited cost as the reason for not proceeding, 1 felt the device was too bulky, and 1 did not provide a reason.

Of the 40 patients who purchased an HFAO, 19 used it regularly for exercise and/or community ambulation, according to their most recent follow-up clinic notes. Eight patients did not have a follow-up appointment within the chart review period, making it unclear whether they were using the device. Six of these patients obtained their HFAO within the last 6 months of the chart access date range and thus would not have been expected to have followed up yet, while 2 had obtained their HFAO earlier.

Thirteen patients who purchased an HFAO were no longer using their device at the time of the most recent encounter within the chart review period. The mean time from the HFAO prescription date to discontinuation was 20 months (range=5-60). Five stopped due to pain or discomfort related to use; 4 found the device too bulky (described as cumbersome to use); 1 had gait difficulties with the device; 1 did not find it helpful; and 2 did not specify a reason. Among the 5 patients who stopped due to pain or discomfort, the HFAO worsened preexisting pain in 3 (2 instances of chronic back pain and 1 of metatarsophalangeal joint pain secondary to osteoarthritis). The other cases of pain or discomfort were thigh hyperesthesia (thought to be due to femoral nerve compression) and uncomfortable pressure over the pelvis. One patient also noted disliking the device’s appearance, though this was not the primary reason for discontinuation.

**Fig. 1:**
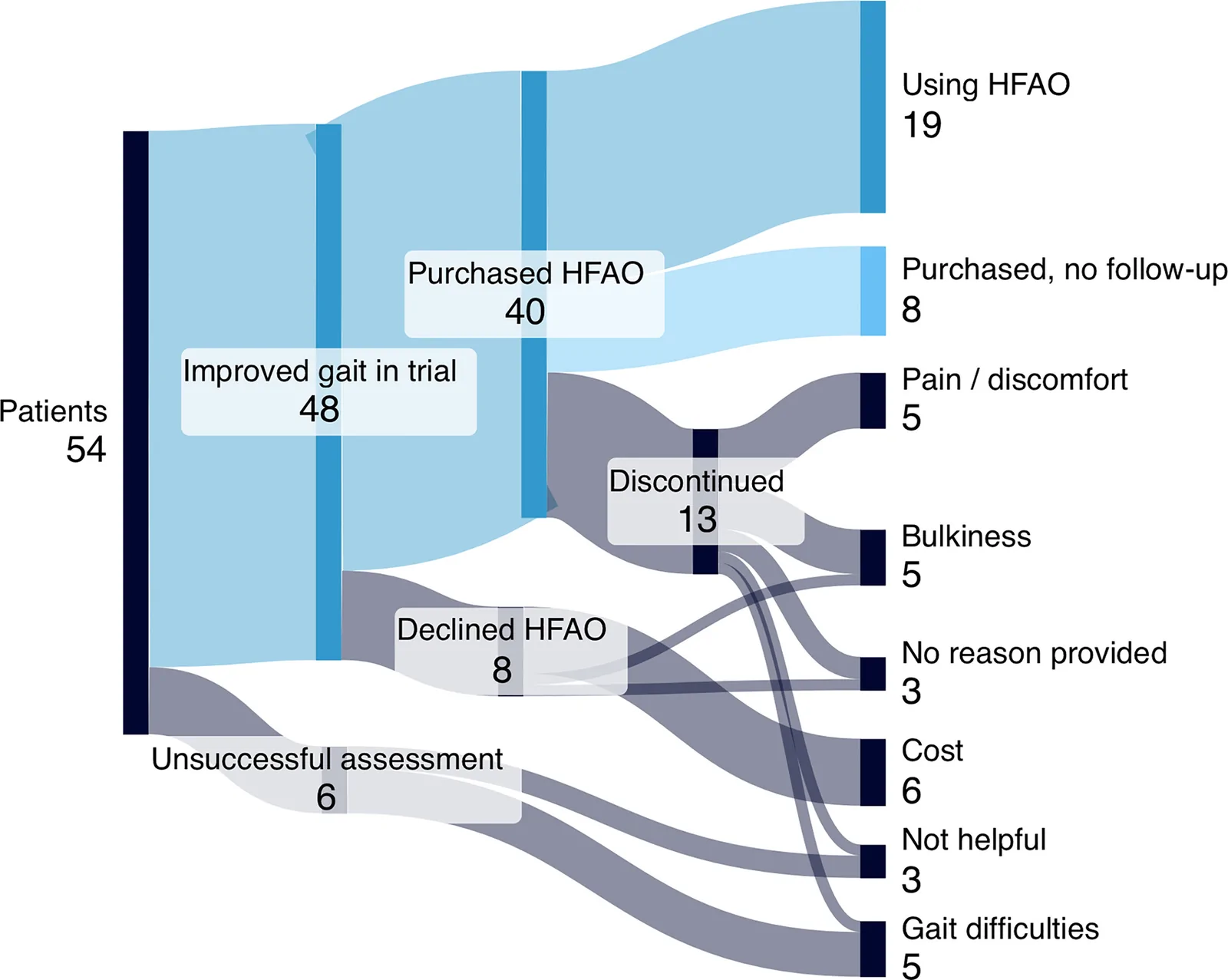
Flow diagram illustrating reasons for not obtaining an HFAO or for discontinuing use.

Table 2 summarizes the above findings and explains why an HFAO was not obtained or discontinued in each category. Figure 1 further illustrates the findings in a flow diagram.

**Table 2:** Reason for not obtaining an HFAO or for discontinuing use

Characeristic	Pain/Discomfort	Gait Difficulties With Device	Bulkiness	Not Helpful	Cost	No Reason Specified
Discontinued	5	1	4	1	0	2
Declined HFAO	0	0	1	0	6	1
Unsuccessful assessment	0	4	0	2	0	0
Total	5	5	5	3	6	3

### Strength and spasticity

Table 3 shows the mean MRC strength scores for hip flexion and knee extension in both limbs, and the mean MAS spasticity scores for each outcome group. Six patients did not have MAS scores in their charts (4 from Using HFAO and 2 from purchased, no follow-up) and were excluded from the spasticity analysis.

**Table 3:** Mean MRC strength scores and MAS spasticity scores across HFAO use groups

Characeristic	Hip Flexion Strength		Knee Extension Strength		Ankle Dorsiflexion Strength		Lower-Limb Spasticity (MAS)
	Weaker Lower Limb	Contralateral Lower Limb	Weaker Lower Limb	Contralateral Lower Limb	Weaker Lower Limb	Contralateral Lower Limb	
Using HFAO	2.2 (1-3)	3.6 (2-5)	3.7 (3-5)	4.1 (3-5)	2.7 (1-5)	3.7 (2-5)	0.77 (0-1+)
Purchased, no follow-up	2.3 (1-4)	3.8 (2-5)	3.6 (2-5)	4.3 (3-5)	2.4 (1-5)	4.0 (2-5)	1.08 (1-1+)
Discontinued	2.0 (1-4)	3.4 (1-5)	3.8 (2-5)	4.3 (3-5)	2.8 (1-5)	3.8 (1-5)	1.50 (0-3)
Declined HFAO	2.5 (1-4)	4.3 (3-5)	3.6 (3-5)	4.5 (3-5)	2.9 (2-4)	4.1 (2-5)	1.19 (1-2)
Unsuccessful assessment	2.7 (2-3)	3.5 (3-5)	3.2 (2-4)	3.3 (3-4)	2.5 (1-4)	3.8 (3-5)	1.33 (1-2)
All groups	2.2 (1-4)	3.7 (1-5)	3.7 (2-5)	4.1 (3-5)	2.7 (1-5)	3.9 (1-5)	1.15 (0-3)
Kruskal-Wallis test statistic	4.26	5.19	2.86	10.5	1.02	2.13	11.0
Kruskal-Wallis *P* value	.371	.268	.581	.033	.906	.711	.026
Significant difference	No	No	No	Yes	No	No	Yes

NOTE. Data are presented as mean (range). A value of 1.5 was assigned to MAS scores of 1+.

Significant differences between at least 2 groups were identified in contralateral knee extension strength and lower-limb MAS scores. Contralateral knee extension strength significantly differed between 2 pairs of groups: Unsuccessful assessment (mean=3.3) vs discontinued (mean=4.3, *P*=.038), and unsuccessful assessment (mean=3.3) vs declined HFAO (mean=4.5, *P*=.015). No significant differences were found for any other strength pairings.

Lower-limb spasticity was significantly higher in the discontinued group (mean MAS=1.5) than in the Using HFAO group (mean MAS=0.77, *P*=.011). No significant differences in MAS scores were found for any other pairings.

**Fig. 2:**
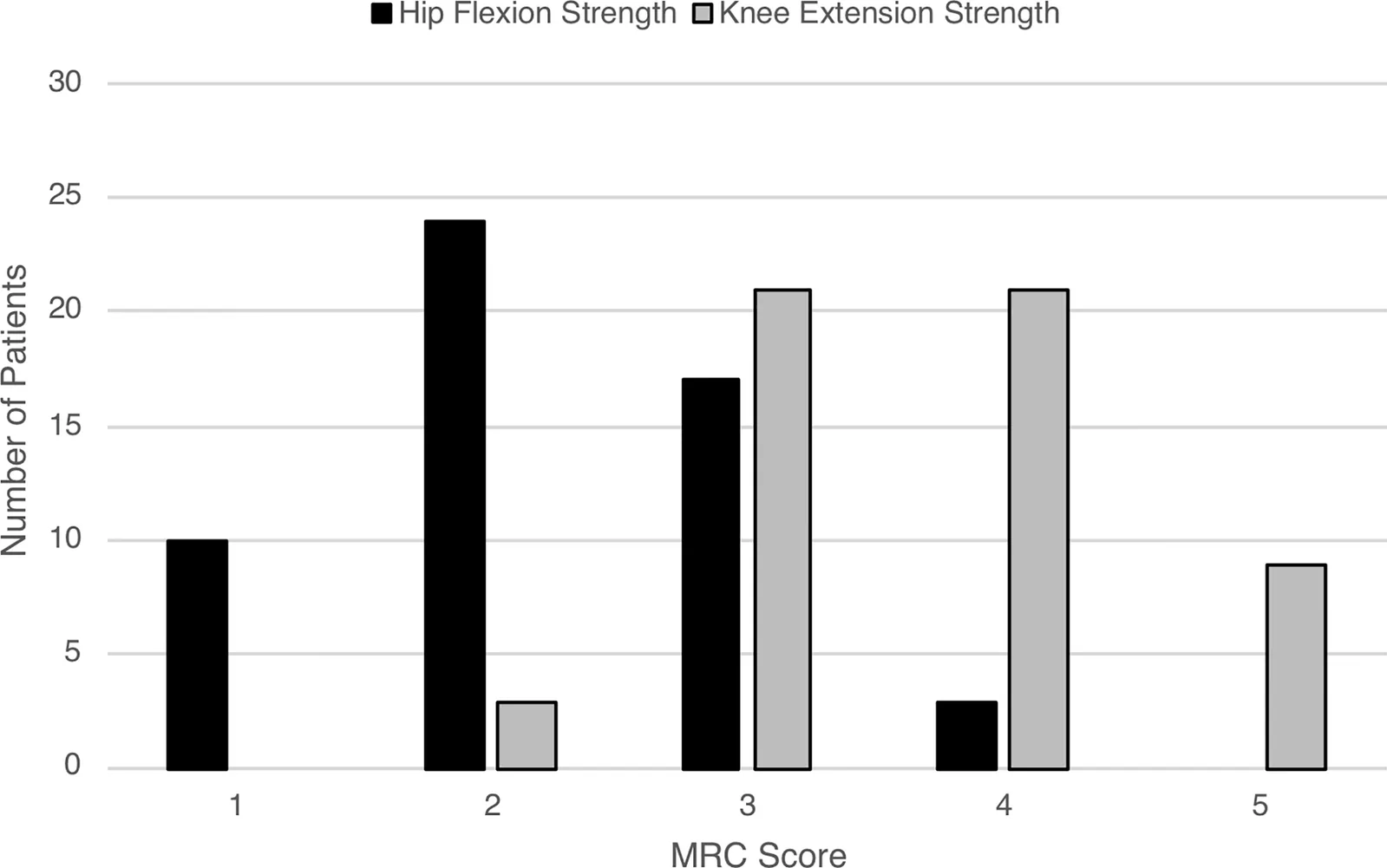
Histogram of weaker side hip flexion and knee extension strength in all patients who underwent HFAO assessment.

Figure 2 shows a histogram of hip flexion and knee extension strength scores for the weaker limb (ie, the prescribed limb in patients with unilateral devices) in all patients who underwent an HFAO assessment. Given the lack of significant differences in hip flexion and knee extension scores between groups, figure 2 illustrates the overall distribution of strength in patients assessed for an HFAO. Figure 2 demonstrates that the distribution of hip flexion strength in the weaker limb approximated a unimodal pattern centered at an MRC score of 2, with most patients having scores between 1 and 3. Knee extension strength was generally higher, clustering around MRC scores 3-4, ranging from 2 to 5. These distributions suggest that patients referred for HFAO assessment commonly had moderate hip flexor weakness with relatively preserved knee extensor function.

## Discussion

This retrospective chart review demonstrates patterns of HFAO use among ambulatory patients with MS at an outpatient physiatry practice. Patients with hip flexor weakness (MRC=1-3) and preserved knee extension strength (MRC≥3) appeared most suitable for HFAO trials. Furthermore, patients who had unsuccessful trials had lower contralateral knee extension strength (mean MRC=3.3, vs. 4.1 or higher for all other groups). Although this difference was not statistically significant across all pairwise comparisons, it may suggest an additional minimum strength criterion for the contralateral side.

Lower-limb spasticity was significantly higher in patients who discontinued HFAO use than in those who used it regularly. All patients with regular HFAO use had a MAS score of 0 or 1, except for 1 patient with a MAS score of 1+. While spasticity does not appear to be an absolute contraindication, this finding suggests it may impede usability or comfort, potentially through difficulty donning the device or increased gait instability.

Pain or discomfort, including exacerbation of preexisting conditions such as low back pain, was the most common reason for discontinuation. These findings underscore the importance of evaluating baseline musculoskeletal complaints and considering how mechanical forces from the HFAO (eg, elastic tension or pressure from the hip belt) may aggravate them.

Device bulkiness and cost were also frequent barriers to use. It is important to discuss these factors with patients during the pre-prescription evaluation, as they may influence a patient's willingness to adopt and persist with an HFAO, even when the device has improved their gait.

These findings highlight the importance of a comprehensive, interdisciplinary patient evaluation that includes muscle strength testing, spasticity grading, gait analysis (both with and without the device), and screening for preexisting pain and comorbidities. An individualized, patient-centered approach is essential when assessing whether the benefits of the device outweigh its drawbacks.

### Study limitations

There are several limitations to this study. Because the study included only patients who underwent an HFAO assessment, no comparisons could be made with patients with MS who were never identified as potential candidates for HFAO. Certain patients may have been implicitly excluded before the assessment stage, including those with significant lower-limb contractures, severe spasticity, or other comorbidities, thereby precluding meaningful HFAO use. As a result, the cohort likely represents a prefiltered population, and the findings may not generalize to all patients with MS and hip flexor weakness. This is further reflected in the high proportion of ADL-independent patients (85%), which was uniform across the outcome groups; whether ADL status influences HFAO outcomes in a more functionally diverse population remains unknown.

Another limitation is the retrospective chart review design, which introduces potential bias and precludes causal inference. Additionally, because the charts were intended for clinical rather than research purposes, certain data were missing or inconsistently recorded. Expanded Disability Status Scale scores were not routinely charted, and spasticity was variably documented, either at the limb level or by individual muscle group. Baseline pain was not systematically documented; it was recorded only when raised as a clinical concern, limiting our ability to evaluate its role in patient selection and outcomes. Several other clinically relevant factors were also not captured, including walking endurance, falls, concurrent use of gait aids or orthoses with the HFAO, joint range of motion, the presence of contractures, daily wear time, and differentiation between HFAO use (community ambulation, in-home use, and exercise). Quantitative gait parameters were not measured during HFAO assessment or follow-up, limiting the ability to objectively characterize changes in gait performance with device use. Follow-up duration varied considerably among patients because it was determined by clinical appointment scheduling rather than a standardized protocol, limiting the comparability of outcomes.

Because all patients were seen by the same physiatrist at a single clinic, the generalizability of the results may be limited, as HFAO use patterns may differ across practices and patient populations. Due to the small number of patients in each analysis group, sex-based analysis was not feasible, which further limits generalizability.

## Conclusions

This retrospective chart review identified patterns of HFAO use among ambulatory patients with MS at an outpatient community physiatry clinic. Many patients benefited from regular HFAO use; however, barriers to adoption and continued use exist, including cost, device bulkiness, and pain or discomfort. Patients with hip flexor weakness (MRC=1-3) and preserved knee extensor strength (MRC≥3) appear most suitable for HFAO trials, and higher lower-limb spasticity (MAS≥2) was associated with discontinuation. These findings provide a foundation for developing clinical prescribing guidelines, though future prospective studies addressing a broader range of factors, including balance, coordination, endurance, and fall frequency before and after device prescription, are needed.

## Disclosures

The authors declare no conflicts of interest and received no funding for this study.
